# Subjective Successful Aging: Factors Related to a Self-Rated Perception

**DOI:** 10.1093/geroni/igaa057.1368

**Published:** 2020-12-16

**Authors:** Marlene Alvarado Rodriguez, Neyda Ma Mendoza-Ruvalcaba

**Affiliations:** University of Guadalajara, Guadalajara, Mexico

## Abstract

Introduction Theoretical successful aging definitions may not consider the older adults perception′s on their own aging. The aim of this study is to analyze factors related to subjective perception of successful aging. Methods: Population based, random sample included n=401 community-dwelling older adults 60-years and older (mean age=72.51,SD=8.11 years,59.4% women). For measurement of subjective successful aging (S-SA), participants were asked to self-rate SA in a Likert scale: “do you believe/feel you are aging well?” Objective Successful aging (O-SA) was operationalized in accordance with Rowe & Kahn definition (no important disease, no disability, physical functioning, cognitive functioning, and being actively engaged). Sociodemographic and health data were also asked. Data were analyzed in SPSSv24. Results: In total 11% were successful agers according to objective measures, while 77.6% rated themselves as successful agers. In the Likert scale of S-SA, specifically 23.4% considered themselves as very much successful agers, 54.1% much, 20.2% somewhat, and only 2.2% said that not at all think they are aging successfully. Education was related to a better perception of S-SA, as well as life satisfaction (p=.000), better subjective health (p=.000), being a spiritual person (p=.000), and not feeling alone (p=.000). Age, marital status, sex, and life-long learning activities were not related to S-SA. Conclusion There is a disparity between subjective and objective successful aging rates Being a successful ager may have a different meaning for each person, and not necessarily involves established criteria. Criteria generated by older adults should be added in theoretical definitions and measures.

